# Electric-Field Fluctuations as the Cause of Spectral Instabilities in Colloidal Quantum Dots

**DOI:** 10.1021/acs.nanolett.3c02318

**Published:** 2023-10-23

**Authors:** Frieder Conradt, Vincent Bezold, Volker Wiechert, Steffen Huber, Stefan Mecking, Alfred Leitenstorfer, Ron Tenne

**Affiliations:** † Department of Physics and Center for Applied Photonics, University of Konstanz, D-78457 Konstanz, Germany; ‡ Chair of Chemical Materials Science, Department of Chemistry, University of Konstanz, D-78457 Konstanz, Germany

**Keywords:** quantum optics, colloidal quantum dots, spectral diffusion, Stark effect, exciton fine structure

## Abstract

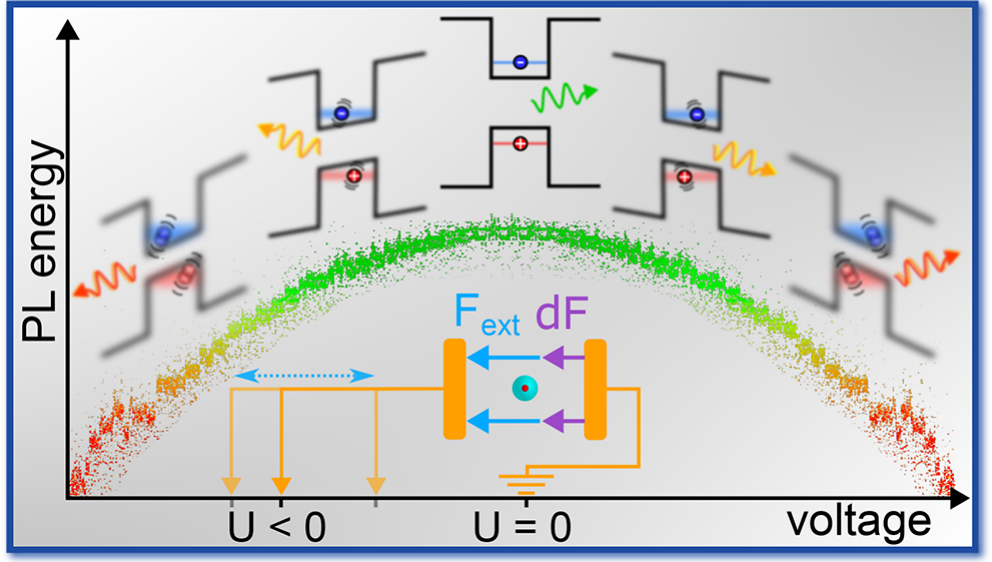

Spectral diffusion (SD) represents a substantial obstacle toward implementation of solid-state quantum emitters as a source of indistinguishable photons. By performing high-resolution emission spectroscopy for individual colloidal quantum dots at cryogenic temperatures, we prove the causal link between the quantum-confined Stark effect and SD. Statistically analyzing the wavelength of emitted photons, we show that increasing the sensitivity of the transition energy to an applied electric field results in amplified spectral fluctuations. This relation is quantitatively fit to a straightforward model, indicating the presence of a stochastic electric field on a microscopic scale, whose standard deviation is 9 kV/cm, on average. The current method will enable the study of SD in multiple types of quantum emitters such as solid-state defects or organic lead halide perovskite quantum dots, for which spectral instability is a critical barrier for applications in quantum sensing.

Over the past three decades, the range of quantum-light sources has greatly expanded. In particular, single-photon or photon-pair emission was demonstrated for a variety of nanosized emitters including organic molecules, solid-state defects, and semiconductor quantum dots (QDs).^1,2^ As such, they form potential building blocks in future quantum-optical technologies, e.g., quantum communication and quantum sensing.^3−6^ While the photon statistics itself is the quantum resource in some applications,^7−11^ most examples rely on quantum interference and thus require coherent radiation and photon indistinguishability.^12−14^

To extend the coherence time in the emission of nanoemitters, they are cooled to cryogenic temperatures to reduce environmental disturbances.^15,1,16,17^ Narrow emission linewidths at low temperatures simultaneously offer new opportunities and challenges. For example, these can be applied to sense fluctuations in the microenvironment of an emitter.^18−20^ The straightforward integration of colloidally synthesized QDs, including the recent emergence of halide perovskite QDs,^21^ into biological settings^22,23^ and semiconductor devices^3,24,25^ offers exciting perspectives for local sensing.^26−28^

However, sensing compels coupling to the environment and therefore typically results in sensitivity to unintentional fluctuations in electric and magnetic fields.^19,29^ In fact, this sensitivity is often implicated with spectral diffusion (SD), i.e., the temporal variance of the energy of emitted and absorbed photons.^30−33^ Such stochastic spectral dynamics currently set considerable limitations on the usability of nanoemitters in quantum applications. Namely, it deteriorates the indistinguishability of emitted photons and hinders the coupling of nanoemitters with cavities, waveguides, and other emitters.^34,35^

The quantitative translation of fluctuations in the electric field to those of the emission spectrum is described by the quantum-confined Stark effect (QCSE). An external electric bias skews the confining potential within the potential well, resulting in a reduction of both the electron and hole quantization energies (see Figure 1a). In a perturbative regime, the resulting red-shift of the exciton transition follows a parabolic dependence on the electric field magnitude^29,36^ (Figure 1b). Therefore, a rapidly changing electric field results in a momentary variation of the emission and absorption lines (Figure 1c,d).

**Figure 1:**
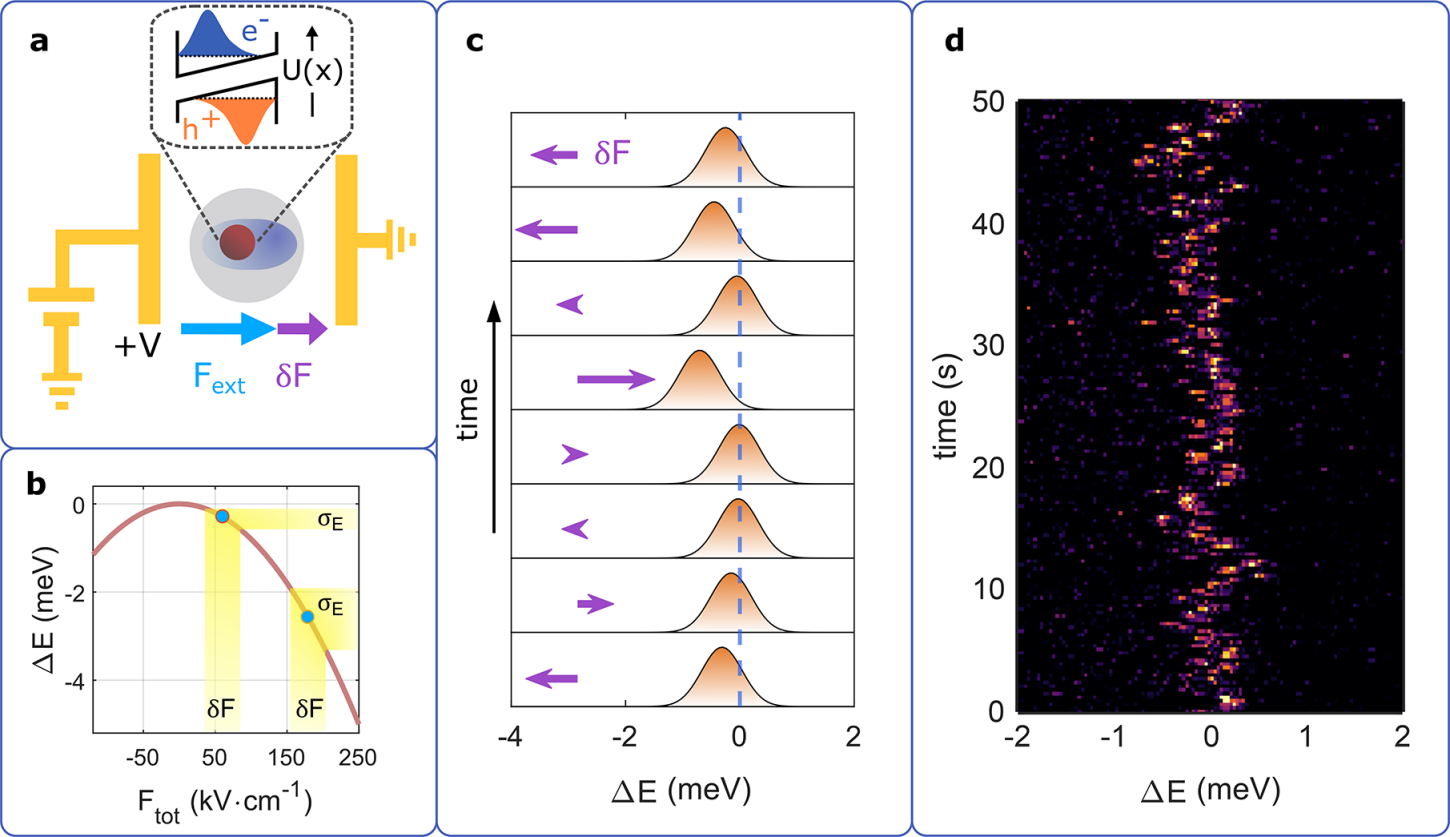
Relation between QCSE and SD. (a) Schematic illustration of the sample: single nanorod placed between two electrodes so that an external field *F*_ext_ can be applied in addition to an intrinsic microscopic field *δF*. The electric bias causes a distortion of the carrier confinement potential and thereby a reduced transition energy (inset). (b) Qualitative example of the red-shift due to QCSE. The energy shift depends quadratically on the electric field. For a larger red-shift, the same δ*F* amplitude induces stronger fluctuations in energy σ_E_ (yellow highlight). (c) Schematic illustration of SD caused by QCSE. Random microscopic field fluctuations (purple arrows) cause an energy redshift that depends quadratically on the field strength. (d) Example data set showing SD. The photoluminescence spectra (each 0.3 s integration time) of a single CdSe/CdS core/shell nanorod at *T* = 6 K exhibit sub-meV irregular fluctuations in energy.

While this causal relation is a prevalent explanation for SD measurements, it is only supported by indirect observations.^37^ For example, the PL linewidth in QDs and nitrogen vacancy centers increases with the electric field magnitude.^38,39^ Indeed, a biasing field, increasing the sensitivity of the spectral line to a fluctuating electric field (Figure 1b), is a reasonable explanation for these observations. However, elevated temperatures or a strengthened exciton–phonon coupling offer alternative explanations.

The current Letter provides the first direct observation of QCSE as the cause of SD in QDs. We show that temporal fluctuations in the PL energy of individual CdSe/CdS dot-in-rod nanoparticles increase the farther away they are driven from the QCSE parabola apex. A straightforward quantitative model for the QCSE matches our results well. Somewhat surprisingly, we find that an inherent dipole exists in many of the nanoparticles studied here. As a result, a significant improvement in the spectral stability can be achieved with a compensating electric field.

In the experiments below, we study SD by measuring photoluminescence from single CdSe/CdS core/shell nanoparticles in a dot-in-rod geometry. The choice of 3.2 nm core size and 20 nm total nanorod length, on average, sets the energy of the fundamental transition to a wavelength around 590 nm (2.1 eV photon energy).^24^ To enhance chemical- and photo-stability, the inorganic nanocrystals are overcoated with a polystyrene/PMMA nanoshell with a thickness of 15 nm. The resulting hybrid organic/inorganic nanoparticles demonstrate superior spectral stability as compared to their uncoated counterparts.^40−42^

A custom-built Er:fiber source generates tunable pulses with subpicosecond duration at a repetition rate of 50 MHz. In order to excite the nanoemitters above the fundamental resonance, the excitation wavelength is set to 540 nm (2.3 eV).^43^ The laser is input into a confocal microscope setup constructed around a bath cryostat (Scientific Magnetics) within which the beam is focused through a 0.9 numerical aperture objective lens (Olympus, M PLAN N, ×100 magnification).^44^ A sample containing the hybrid nanoparticles embedded into microcapacitor structures with a 2 μm gap on a SiO_2_ substrate (see further details in Supplementary Notes 1–3) is placed in the focal plane of the microscope and held at a temperature of 8 K. The PL collected through the same objective lens is spectrally resolved with a grating spectrometer (PI Acton, SP2300, 2400 lines/mm grating) and detected by an electron-multiplying charge-coupled device (EMCCD) camera (Andor, Newton 970). A typical time trace of PL spectra is presented in Figure 2a with the energy axis centered around the brightest emission line at 2.125 eV. Following previous reports, the three narrow emission lines can be attributed to the F, A_1_, and A_2_ transitions in a neutral QD. The various peaks arise from the fine-structure splitting of the exciton ground state according to the discrete projections of the angular momentum on the main axis of the nanorod (*m*_*f*_).^45,46^ The weakest and lowest-energy peak (F, around −1 meV) originates from the dipole-forbidden recombination of the *m*_*f*_ = 0 exciton state.^45,46^ The two additional spectral peaks (A_1_ and A_2_) result from a lift of the degenerate *m*_*f*_ = ±1, yielding two dipole-allowed transitions with orthogonal transition-dipole moments.^44,47^ Within the 90 s acquisition time, the three spectral lines fluctuate synchronously within a range of approximately 1.5 meV, indicating that they originate from a single quantum emitter. Integrating over the time axis in Figure 2a (top inset), all spectral features are significantly blurred, in contrast to the individual 1 s exposure measurements.

**Figure 2:**
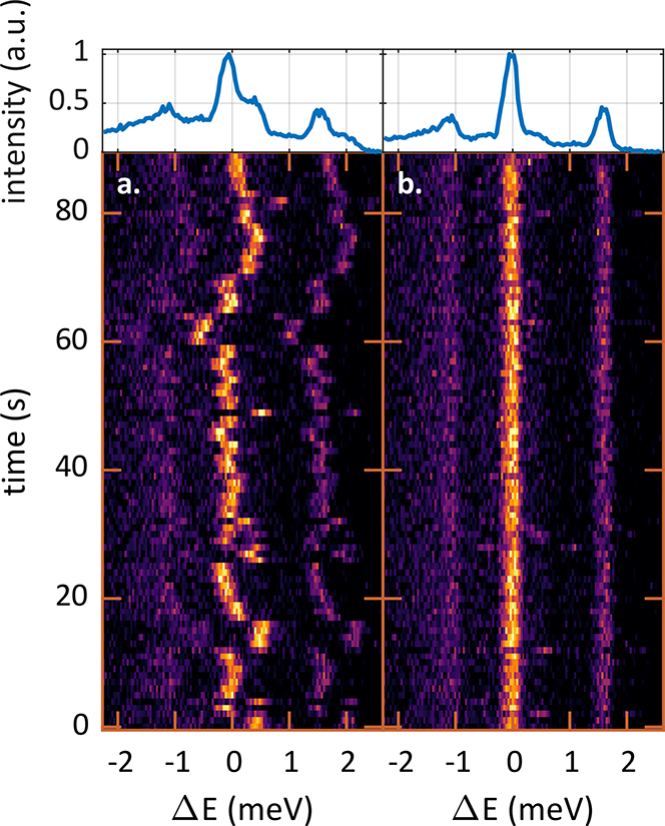
Correcting SD in postprocessing. (a) 90 consecutive 1 s acquisitions of a single QD PL spectrum. Horizontal shifts between the spectra manifest the SD. The top inset presents the integrated spectrum from all measurements. (b) The same data set corrected in postprocessing with the cross-correlation algorithm. Spectra are now well aligned on the energy axis. The integrated spectrum (top) presents three narrow lines, indicating radiative transitions from the excited states to the ground state.

Alternatively, spectral shifts can be evaluated in postprocessing in order to realign the spectra with one another (Figure 2b). To this end, a cross-correlation-based algorithm, robust to the naturally low signal-to-noise ratio (SNR) of the spectra, is applied.^44^ Subsequently, the three emission lines are clearly aligned in energy. In the integrated spectrum, the linewidth of each feature is similar to that of individual measurements, i.e., less than 0.3 meV. A single-spectrum acquisition time of 1 s is selected to obtain a reasonable SNR for the correction algorithm while enabling sufficient sampling of the transition-energy dynamics. This procedure provides quantitative information about spectral fluctuations and is used below to statistically analyze SD.

The parabolic dependence of the transition energies on an applied electric field means that both the PL wavelength and its sensitivity to field perturbations vary with an external bias. In the regime of small field fluctuations, energy shifts are proportional to the first derivative of the QCSE parabola. As a result, assuming an equal amplitude of field fluctuations (δ*F*), the range of energy shifts increases with an electric-field-induced red-shift of the PL (yellow highlight areas in Figure 1b).

Applying an adjustable DC voltage between −100 and +100 V, the electric field in the *z* direction is tuned between −500 and +500 kV/cm, respectively. Within this range, the PL energy shifts by up to 26 meV, roughly 100 times the spectral linewidth. In contrast to previous reports of nearly complete darkening of the emission,^38,48^ even under the relatively strong fields used here, the spectrally integrated PL intensity reduces by 40%, at most. Because of the dielectric nature of the exciton’s environment (CdSe, CdS, and polymer encapsulation), the local electric field strength is reduced to approximately 0.35 of the field amplitude in the surrounding vacuum^49^ (see Supplementary Note 4). In the current text and contained graphics, we use only the nominal values of the electric field, i.e., the field amplitude in a vacuum.

An initial electric-field sweep is performed to determine the PL peak energy as a function of the external electric field (28 voltage steps, 1 s acquisition, each). Figure 3a shows the relative energy shift (Δ*E*) as a function of the applied electric field (*F*_ext_) in a range from −400 to +350 kV/cm (blue squares). The field dependence of the energy redshift fits to a parabolic function to a rather high degree (Figure 3a, orange line)
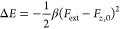
1where *F*_*z*__,0_ is the projection of the inherent electric bias of the QD on the *z*-axis, defined by the direction of the external field. Such a built-in bias was previously reported for CdSe core-only and CdSe/CdS core/shell QDs in both room-temperature and cryogenic measurements.^29,36,38,48,50^ It is assigned to a break of centrosymmetry in the exciton state, likely due to a combination of asymmetry in the confining potential and strain distribution.^36,51^ In the measurement shown in Figure 3a, the apex offset *F*_*z*__,0_ is estimated to be 20 kV/cm. We also note that beyond the perturbative regime, for an asymmetric system, the parabolic approximation breaks altogether.^48^ However, our measurements did not find any evidence of such nonparabolic trends for the CdSe/CdS core/shell nanorods used in this work.

**Figure 3:**
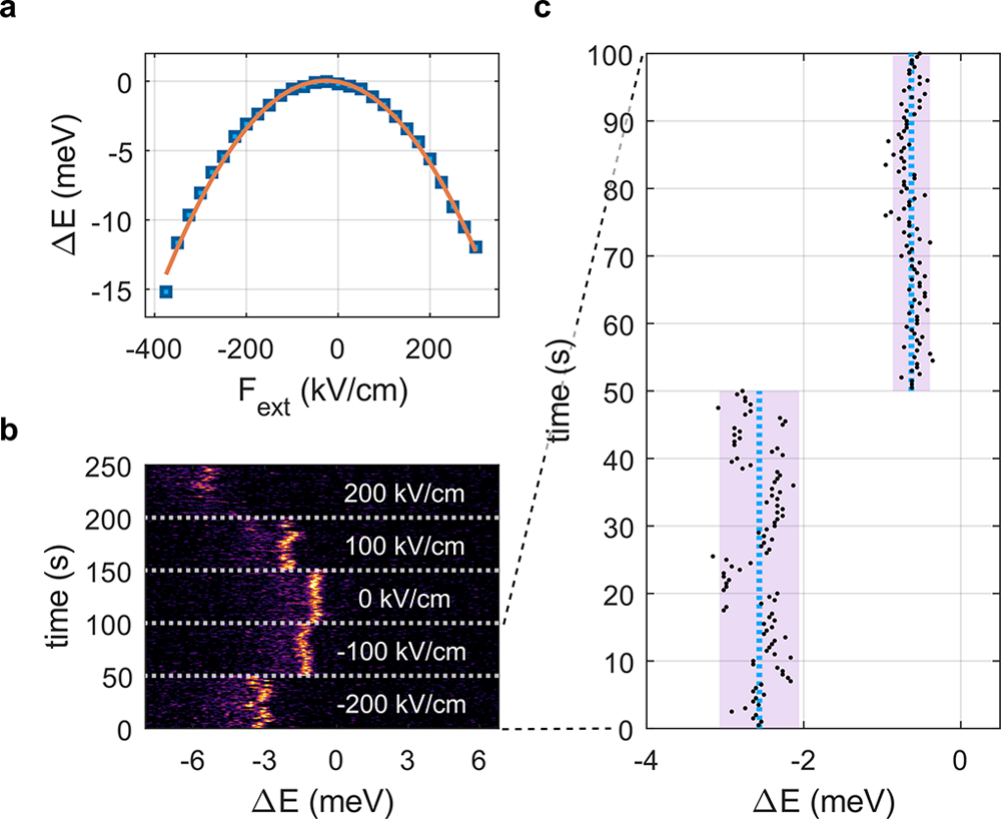
Joint measurement scheme for the QCSE and SD. (a) The energy shift of the brightest spectral peak vs nominal field amplitude for a single nanoparticle (blue squares). A parabolic fit is overlaid in orange. (b) Consecutive PL spectra (0.5 s acquisition time) taken for the same QD. The applied external field is changed every 50 s (white dashed lines; field value given alongside). (c) Momentary energy shift analyzed for the first 100 s of the measurement in (b). Light blue dashed lines indicate the mean energy shift, and violet rectangles display the region of two standard deviations to each side of the mean.

To analyze the interplay between the applied field and emission energy dynamics, spectral time traces (100 spectra, 0.5–1 s acquisition time) are taken under several values of the applied field. Figure 3b presents such a time trace, divided into five sections according to the external bias (dashed white lines). With increasing red-shift of the emission line, SD fluctuations clearly intensify in range. For a quantitative evaluation of SD, the peak energy shift in each spectrum is determined via the cross-correlation algorithm. An example of the results, for two temporal sections, is shown in Figure 3c where the external field is switched from −200 to −100 kV/cm at *t* = 50 s. In each section, the average energy is marked with a blue dashed line, and a violet area represents the standard deviation. Indeed, with a blue-shift of 2 meV, the standard deviation in the second section is smaller by a factor of 2 compared to that of the first section.

Figure 4 demonstrates this effect for two individual QDs, labeled as QD1 and QD2 from this point onward. The PL emission energy as a function of the applied electric field, analyzed from a voltage-sweep measurement, is shown in Figures 4a and 4b for QD1 and QD2, respectively. As expected, the dependence follows a parabolic trend (eq 1) depicted by the orange lines. Notably, the intrinsic offset field parameter, *F*_*z*__,0_, is positive and large (+520 kV/cm) for QD1 while negative and small (−27 kV/cm) for QD2.

**Figure 4:**
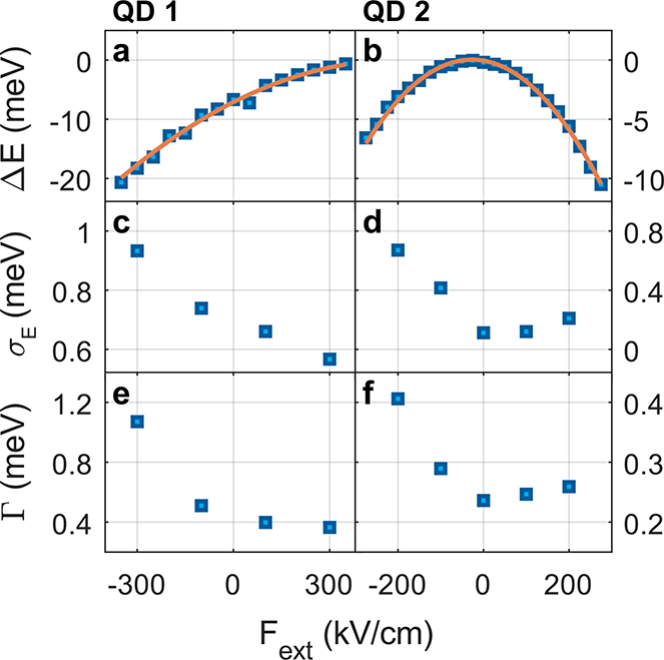
SD measurements for two individual nanoparticles. The energy shift of the PL emission versus the applied electric field for (a) QD1 and (b) QD2 was analyzed from an electric-field-scan measurement. Orange lines are a parabolic fit for the QCSE following eq 1. The standard deviation of PL emission peak energy was analyzed from a time series of spectra, as shown in Figure 3b, for (c) QD1 and (d) QD2. Dependence of the spectral width of the emission line, after correction for slow SD, is dependent on the external electric field for (e) QD1 and (f) QD2.

To analyze SD, we conceptually divide the time scale of fluctuations into two regimes: slow dynamics that can be tracked with the 1 s sampling time of our measurement and fast fluctuations which occur within each acquisition. The observable fluctuations in Figures 3b and 3c belong to the former, whereas the latter manifests as a broadening of the emission line. Figures 4c and 4d depict the standard deviation of the emission peak energy (σ_*E*_) versus the applied electric field for QD1 and QD2, respectively. For both QDs, the standard deviation, a simple quantitative measure of slow spectral fluctuations, increases with an energy redshift of the PL, that is, with larger Δ*F* = |*F*_ext_ – *F*_*z*__,0_|. Importantly, the point of maximal spectral stability is aligned with the apex of the QCSE parabola (*F*_*z*__,0_), a good indication of the relation between SD and QCSE. In fact, these results qualitatively confirm the picture presented in Figure 1b—the range of spectral fluctuations increases with further offset from the apex of the parabola. Supplementary Figure S4 shows similar analyses for two additional nanoparticles that confirm this conclusion.

We note that to perform the spectral diffusion analysis as a function of an external electric field, we postselect measurements that fulfill several criteria. First, as the spectral correlation algorithm relies on sufficient SNR, spectra with a particularly low signal level are dismissed. Second, as these measurements require approximately 5 min of laser exposure time, a portion of the measurements were discarded due to photobleaching. Finally, the measured QCSE shift must exhibit a substantial nonlinear dependence to vary the sensitivity of the PL energy to field fluctuations. Further details regarding data postprocessing and analysis are given in Supplementary Note 5. Importantly, none of the measurements featuring a statistically significant result displayed a contradictory trend to that shown in Figures 3–5 (and Figures S4 and S7). To further establish a relation between QCSE and the variation of spectral fluctuations, Figure S5 presents three data sets in which the PL spectrum of a QD is measured during a sweep of the electric-field amplitude. In all three cases, the spread of energy shifts around the average values clearly increases with a larger Δ*E*.

**Figure 5:**
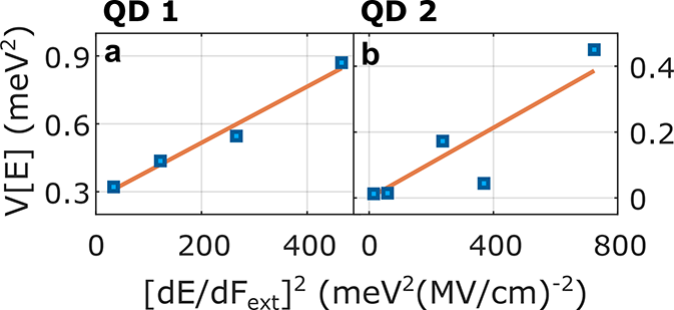
A quantitative analysis of the relation between SD and QCSE. The dependence of slow fluctuations variance (σ_*E*_^2^) on the square of the derivative of the energy with respect to the electric field for (a) QD1 and (b) QD2. Linear fits (orange lines) indicate that the model presented in eq 5 is in good agreement with our results for parameter values.  (QD1) and  (QD2).

A related topic is the 3D orientation of the CdSe/CdS nanorod with respect to the electric field. In this work, the orientation of the hybrid nanoparticles is not controlled nor is it measured. Correspondingly, we observed a large variation in the QCSE response of individual nanorods. We note that the measurement of the two nanorods presented in Figure 4 (and the two shown in Figure S4) present a rather strong QCSE response (large β). Therefore, their long axes are likely oriented nearly parallel to the external electric field. Under the model introduced in Figure 1, the impact of electric field fluctuations is also the strongest in this direction. Thus, It is reasonable that the effect of the electric field on spectral fluctuations is most evident for these nanorods.

In an alternative analysis, focused on fast fluctuations, we align the consecutive spectra within each field magnitude section and obtain an average spectrum with a high SNR (see Figure S6). In order to characterize the emission line shape, we fit each averaged spectrum with a Gaussian function
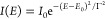
2where *I*(*E*) is the PL spectrum, *E*_0_ the energy peak, and Γ the observed spectral linewidth. Figures 4e and 4f present Γ as a function of the external field magnitude for QD1 and QD2, respectively. Similarly to σ_*E*_, the linewidth decreases when tuning the external field toward the apex of the QCSE parabola (*F*_*z*__,0_). For a substantial portion of our measurements, the presence of a large inherent bias means that the linewidth can be significantly narrowed by the application of an external field. For QD1, for example, Γ at the apex is three times smaller than that under the maximally negative external field.

Indeed, previous studies already pointed out this relation for both QDs and nitrogen-vacancy centers in diamond, supporting a connection between QCSE and SD.^38,39^ However, the data shown in Figures 4c and 4d provide a more direct observation—a correlation between spectral dynamics and the applied field. That is, we observe the energetic dynamics itself rather than its consequence on a static measurement. A few alternative explanations can account for the broadening of PL emission lines in a steady state, such as thermal effects and enhanced phonon coupling. These cannot provide an explanation for the extended range of slow spectral fluctuations shown in this work.

To further support the mechanistic explanation for SD described above, we compare the results of Figure 4c,d to those of a straightforward statistical model. Including a local fluctuating field *δF⃗*(*t*) in eq 1, the PL transition energy as a function of time is expressed as
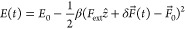
3The field fluctuations can be divided into parallel and orthogonal axes δ*F⃗* = δ*F⃗*_⊥_ + δ*F*_*z*_·*ẑ* with respect to the applied field. Assuming that fluctuations in orthogonal axes are independent of one another
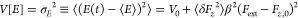
4where *V*_0_ consists of terms that contribute to the variance but do not depend on the strength of the external electric field, *F*_ext_ (see Supplementary Note 6 for the full derivation). In order to fit this model to our data, we note that [d*E*/d*F*_ext_]^2^ = β^2^(*F*_ext_ – *F*_*z*,0_)^2^ which allows us to rewrite eq 4 as
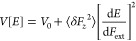
5Intuitively, eq 5 merely reflects the fact that spectral fluctuations are proportional to the absolute value of the derivative (d*E*/d*F*_ext_), and therefore the variance increases with its second power.

In Figures 5a and 5b the same data as in Figures 4c and 4d are plotted against [d*E*/d*F*_ext_ ]^2^, respectively. Two additional data sets are presented in Figure S7. Orange lines portray the linear fits of the data according to eq 5. From these, we obtain a unique estimate for the standard deviation of the local electric field averaged over the four measurements presented in the paper and Supporting Information, 9 ± 2.5 kV/cm. We note that this value reflects field amplitude within the CdSe core of the nanoparticle–multiplied by a factor of 0.35 due to the dielectric environment (see Supplementary Note 5). The very similar ranges for δ*F*_*z*_ for all analyzed nanoparticles strengthen the validity of the simplified general model applied here.

To explore the cause of the microscopic electric-field fluctuations measured in this work, it is important to inspect the role of the excitation laser itself. The absorption of a photon introduces excess energy that disperses through the lattice of the QD and its surrounding. Thus, it is reasonable to expect that the laser can instigate charge dynamics around the core of the nanorod, which cause transient electric fields. However, measurements of the extent of spectral fluctuations against the laser excitation power and wavelength display insensitivity to both parameters (see Supplementary Note 7). This fact indicates that the electric-field fluctuations detected here are inherent to the hybrid nanoparticles and/or their microenvironment (e.g., substrate). This observation guides the direction of future studies toward variations in the synthesis and sample preparation and provides a positive outlook toward improving the spectral stability of these emitters.

To summarize, the current work provides the first direct observation that photon energy fluctuations in the PL of individual colloidal QDs result from spurious electric fields in their microenvironment. Applying an external field, we find that the photon energy variance increases with an energy redshift of the emission line. A quantitative fit of this dependence to a straightforward model estimates the standard deviation of the electric field sensed by the exciton as 9 kV/cm, on average.

A continuous improvement in spectral stability is a crucial step toward the application of semiconductor nanocrystals as local quantum sensors of their environment. A direct consequence of this study is that the spectral stability of single QDs, often biased by an inherent field, can be substantially improved by applying an inverse unbiasing field. To further improve spectral stability, the method presented here can provide spectroscopic feedback for the optimization of the synthesis and sample preparation procedures for quantum applications. Performing such studies with a higher temporal and spatial resolution, taking advantage of new detector technology^10^ and nanocrystal architectures,^52,53^ could play an important role in isolating the microscopic source of electric field disturbances.

## Supporting Information Available

Details about nanocrystal synthesis, sample preparation, data processing and mathematical modeling (PDF)
