# Supplementary material for: Electric-Field Fluctuations as the Cause of Spectral Instabilities in Colloidal Quantum Dots
Source: Nano Lett. 2023 Oct 23;23(21):9753–9. doi: 10.1021/acs.nanolett.3c02318 (PMC10636921; doi:10.1021/acs.nanolett.3c02318)
Supplement: Supplementary file 1 — nl3c02318_si_001.pdf [file nl3c02318_si_001.pdf]

Supporting information for "Electric-field fluctuations as the cause of spectral instabilities in colloidal quantum dots"

Frieder Conradt[1,‖], Vincent Bezold[1,‖], Volker Wiechert[1], Steffen Huber[2], Stefan Mecking[2], Alfred Leitenstorfer[1,*], Ron Tenne[1,*]

[1] Department of Physics and Center for Applied Photonics, University of Konstanz, D-78457 Konstanz, Germany

[2] Chair of Chemical Materials Science, Department of Chemistry, University of Konstanz, D-78457 Konstanz, Germany

[*] Email: ron.tenne@uni-konstanz.de

[*] Email: alfred.leitenstorfer@uni-konstanz.de

Supplementary note 1: Capacitor Preparation

To apply an external electric field on single nanocrystals, we prepare an interdigitated gold-electrode structure with a gap size of 2 µm. The electrode structure is fabricated via electron-beam lithography (EBL) on a crystalline SiO_2 substrate. To do so, the substrate is coated with a 220 nm layer of PMMA (A4) resist and a 5 nm aluminum layer to prior to the patterning. Developer fluid is a mixture of isopropanol, MIBK methyl isobutyl ketone (MIBK) and methyl ethyl ketone (MEK) (ratio: 3:1:0.06) in which the exposed sample is immersed for 60 seconds. Subsequently, a layer of 5 nm Chromium (Cr) for enhanced adhesion and 60 nm of gold are deposited on the developed sample by thermal evaporation. By placing the sample for 12 hours or more in an acetone bath, the remaining resist and the corresponding metal layer is lifted off. The sample with the remaining electrodes is mounted on a circuit board and connected with bond wires. The gap size was chosen to enable the application of a strong field magnitude of up to 500 kV/cm using a reasonable analog voltage of 100 V. An optical microscope image of the final structure is shown in Fig. S1.

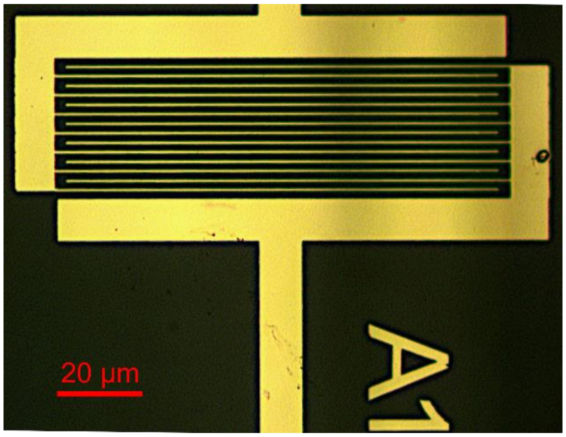

Fig. S1. An optical-microscope image (edited) of a gold electrode structure on a SiO_2 substrate. The structure is fabricated with electron-beam lithography. The gap between adjacent metallic strips size is about 2 µm.

The precise gap size is measured with an atomic force microscope (AFM). Such a measurement is shown in Fig. S2. The average gap size if 1.85(5) µm.

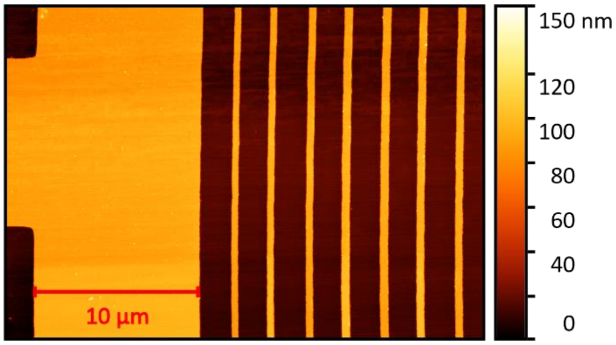

Fig. S2. Atomic-force-microscope (AFM) image of a capacitor structure (same as in Fig. S1). The average gap size is of 1.85(5) µm.

Supplementary note 2: Quantum dots deposition

Embedding quantum dots (QDs) into the capacitor structure is done by a drop-casting process. Prior to this, the sample is placed in a plasma cleaner to reduce droplet formation during drop casting. A water-based dispersion of hybrid nanoparticles (see Supplementary note 3) is diluted into ethanol in a ratio of 1:50. A 5 µl drop of this solution is then deposited on the sample. For some samples, this step was repeated to increase the concentration of nanoemitters. The ethanol solvent supports the formation of a thin liquid film rather than water droplets and leads to a faster evaporation which guarantees an even distribution of the nanoparticles. The distribution of functional nanoparticles is verified with a photoluminescence (PL) imaging microscope. Fig. S3 shows a capacitor structure with embedded nanoemitters. Backlight illumination enables the observation of both the structure (dark portions) and the photoluminescence of the QDs. In Fig. S3, the digital saturation of the PL signal is done intentionally to highlight the dimmer scattered light from the substrate surface.

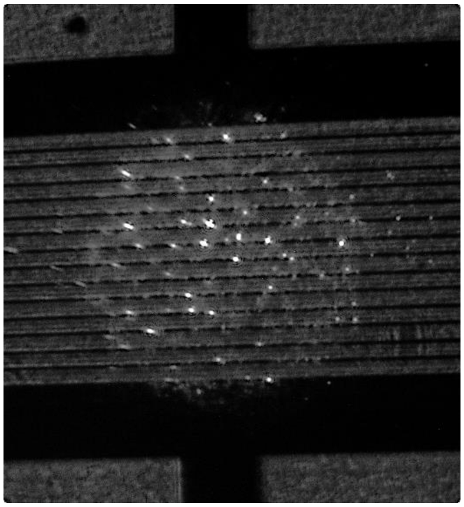

Fig. S3. PL of nanoemitters embedded in a capacitor structure. Due to backlight illumination, the metallic structure appears dark while scattered PL light from the substrate surface exposes nonmetallic portions. The image not only shows that QDs are embedded within an electrode gap but also helps to ensure that the emitters do not cluster and can be observed individually.

Supplementary Note 3: Synthesis of encapsulated nanorods

Full details regarding the synthesis of nanorods (NRs) used the reader is referred to reference 1. In this section, we provide a summary of this procedure.

The following materials were used in the synthesis: Cadmium oxide (> 99.99%-Cd, lot MKBT7524V), hexylphosphonic acid (95%, lot MKBX1133V), oleylamine (70%), oleic acid (90%), octanethiol (> 98.5%), tri-*n*-octylphosphine (TOP; 99%), tri-*n*-octylphosphine oxide (TOPO; 99%), methyl methacrylate (MMA) (99%, contains < 30 ppm MEHQ as inhibitor), *p*-divinylbenzene (85%, stabilized with 4-*tert*-butylpyrocatechol), styrene (> 99%, contains 4-tert-butylcatechol as stabilizer), 2,2'-azobis(2-methylpropionitrile) (98%) and sodium dodecyl sulfate (> 99%, dust free pellets) and octadecene (90%) were purchased from Sigma-Aldrich. *n*-Octadecylphosphonic (ODPA, 98%, lot 807601N16) acid was obtained from PCI.

Standard organic solvents and chemicals were obtained from various commercial suppliers such as Sigma-Aldrich, ABCR, VWR and Roth.

Deionized water was distilled under a nitrogen atmosphere. Methyl methacrylate was filtered over basic aluminum oxide, dried over 4 Å molecular sieves, degassed by three freeze-pump-thaw cycles and stored inside a glovebox at -30 °C. Styrene was vacuum transferred, degassed by three freeze-pump-thaw cycles and stored inside a glovebox at -30 °C.

Synthesis of CdSe cores

The synthesis of the CdSe core particles follows a modified protocol by Carbone *et al.*[2]. Shortly, 60 mg of CdO, 280 mg of octadecylphosphonic acid and 3000 mg of tri-n-octylphosphine oxide (TOPO) were mixed in a three-neck flask. Under vacuum and at a temperature of 150°C the reagents were degassed for one hour. Under a nitrogen environment, the temperature was increased to 330°C and the solution was stirred for around two hours. The mixture was stirred until it turned clear, indicating the complexation of the Cd^{2+} ions. Subsequently 1.8 mL of tri-n-octylphosphine (TOP) was added and the mixture heated up to 370°C. A TOP-Se solution is prepared by 57 mg of Se and 360 mg of tri-n-octylphosphine by stirring for one hour at room temperature. This solution is then injected, and the reaction cooled down as soon as the desired size of the CdSe seeds is reached. The typical growth time is about 30 to 60 seconds. An amount of 3 mL of toluene was added at a temperature of 100 °C. Afterwards the cores were purified by precipitation in methanol, centrifugation, discarding the supernatant and redispersion in toluene. This is repeated three times. Finally, the cores were separated, dried and redispersed in 4 mL of *n*-hexane.

Synthesis of CdSe/CdS/CdS rods

CdSe/CdS seeded nanorods were prepared following the synthesis procedure published by Carbone *et al.* and upscaled by a factor of 1.5[2]. In a following step, a second overcoat shell of CdS was added, realizing CdSe/CdS/CdS core/shell/shell. The recipe for this synthesis was adapted from the one described by Coropceanu *et al.*[3] which uses the seeded CdSe/CdS nanorods as its starting point.

20 nmol of the CdSe/CdS rods in hexane are mixed with 1.5 mL of octadecene, 1.5 mL of oleylamine and 1.5 mL of oleic acid. Residues of water and hexane were removed for 45 minutes at a temperature of 50°C and 15 minutes at 105°C in vacuum. The final reaction is realized at a temperature 310°C and under a nitrogen atmosphere. The injection of the precursor solutions (3 mL of Cd-oleate in octadecene and 3 mL of 1-octanethiol in octadecene) already started when reaching 210°C. Both solutions were added at a rate of 1.5 mL/h (theoretically 1 monolayer of CdS per hour). The desired shell thickness (here: 2 mono layers)

and the size of the initial CdSe/CdS NRs define the amount of precursor solution. Assuming a complete conversion of the Cd-oleate, the ratio of octanethiol to Cd was chosen to be 1.2:1. After the precursor was added, the reaction was cooled down and precipitated in acetone/MeOH (70:30). The NRs were extracted via centrifugation, dried, and redispersed in toluene.σ

<u>Polymer encapsulation of nanorods</u>

In the next synthetic step, the NRs were embedded into cross-linked polystyrene spheres overcoated with an additional cross-linked polymethyl methacrylate (PMMA) shell. The polystyrene encapsulation is based on a procedure by De San Luis *et al.*[4] . The aqueous phase is prepared with 42 mg of sodium dodecyl sulfate (SDS) and 21 mg of $NaHCO_3$, both degassed and dissolved in 25 mL of distilled water. The organic phase consists of 2.3 mL of styrene, 84 µL of *n*-hexadecane, 21 µL of 1,4-divinylbenzene and 0.6 mL (5 nmol) of the NRs dispersed in toluene. After stirring, the organic phase is added to the aqueous phase. A miniemulsion was prepared by ultrasonication for 4 minutes at 80% intensity, while the mixture was cooled in an ice batch to prevent polymerization. A mixture of 21 mg of SDS and 10 mL of distilled water was added afterwards to the emulsion, which was subsequently heated up to 75 °C. Polymerization was initiated by adding 11 mg of potassium peroxydisulfate, dissolved in 4 mL of distilled water. The polymerization process lasted for 6 hours.

In a final step, an additional cross-linked PMMA shell (theoretical thickness of 15 nm) was added to coat the nanoparticles. For this purpose, 2 mg of AIBN were added to 20 mL of the polystyrene dispersion After heating up the mixture to 75°C, methyl methacrylate (MMA) mixed with 1,4-divinylbenzene (100:1 weight ratio) was added via a syringe pump with an injection rate of 0.25 mL/min. The mixture was stirred for 3 hours. The required quantity of MMA was calculated by the solids content of the dispersion and size (DLS number average) of the initial particles and the desired shell thickness assuming the polystyrene particles to have a density of 1.05 g/mL.

Supplementary note 4: Estimation of the effective electric field

Due to the permittivity of the polystyrene and CdS shells, the effectively electric field sensed by the exciton, confined to the core, is a fraction of the applied field strength $E_a = \frac{U_a}{d}$ in vacuum with voltage bias U_a and gap size d. The effective electric field in a dielectric sphere placed in an external field can be estimated as[6]

$$E_{eff} = \frac{3 \cdot \varepsilon_{gap}}{\varepsilon_{sphere} + 2 \cdot \ \varepsilon_{gap}}. \qquad \text{S1}$$

We obtained a value of $E_{QD} = 0.35 \cdot E_{applied}$ using $\varepsilon_{PS} = 2.5$ and $\varepsilon_{CdS} \approx 9.2$[9] as the value of permittivity for polystyrene and CdS, respectively. Here, the effect of the small CdSe core is neglected as its permittivity ($\varepsilon_{CdSe} \approx 8.5$) nearly matches that of the CdS shell.

Supplementary Fig. S4: Spectral diffusion under an external electric field for two additional QDs

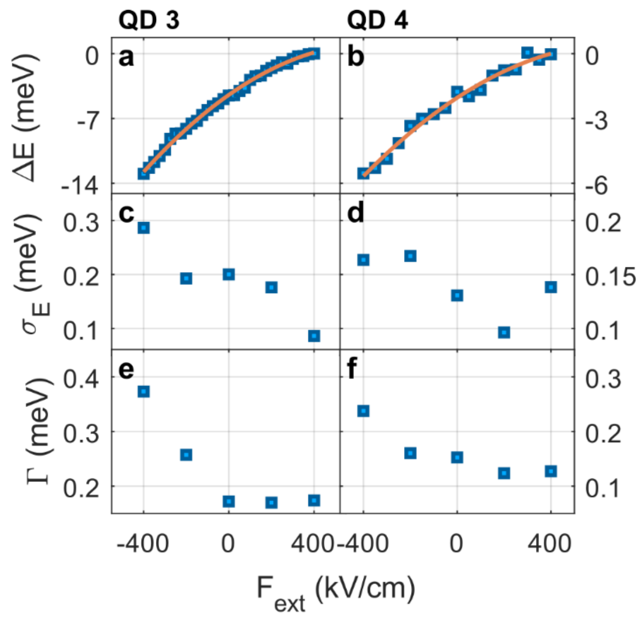

Fig. S4. Spectral diffusion versus electric field for two additional individual QDs beyond the data presented in the main text. This figure is identical in format to Fig. 4 of the main text. The energy shift of the PL emission versus the applied electric field for (a) QD3 and (b) QD4 analyzed from an electric field scan measurement. Orange lines are a parabolic fit for the QCSE. The standard deviation of PL-emission peak energy analyzed from a time series of spectra, as shown in Fig. 3b, for (c) QD3 and (d) QD4. Dependence of the spectral width of the emission line, after correction for slow SD, on the external electric field for (e) QD3 and (f) QD4.

Supplementary Note 5: Preprocessing of measurement data

The principal challenge to quantify possibly fast spectral fluctuations is to precisely measure shifts of spectra despite a relatively low per-pixel signal-to-noise ratio (SNR). The method used here is based on a cross-correlation algorithm of N_{spec} consecutive spectra with ~1 s acquisition time each[5] and is similar to algorithms used to obtain high quality images in astrophotography (lucky imaging). Even though PL emission at cryogenic temperatures is significantly more stable in comparison with room temperature luminescence, the presence of intensity fluctuations still reduces the performance of the correlation algorithm. Therefore, in an initial step, we filter out spectra in which the total intensity is more than three standard deviations below the mean over all spectra.

Additional preprocessing steps mitigate the effect of spectral instabilities that are not attributed to spectral diffusion, such as cosmic rays and charging. Single spectra whose peaks shift by more than three standard deviations from the mean peak position are excluded from further analysis. We note, however, that such events are hardly present in the data used in this paper.

On rare occasions, irreversible sudden spectral jumps are observed. Since we suspect that such sudden shifts are due to, e.g., changes in the chemical environment of the nano particle rather than SD, we disregard these in the analysis of spectral shifts. To identify sudden and large spectral jumps, the vector of spectral positions is convoluted with a step function:

$$\vec{S} = \vec{a} * \Delta\vec{E}, \qquad \text{S2}$$

where $\vec{a}$ is defined as $\vec{a} = (-1, -1, \dots, -1, 1, 1, \dots, 1)$ with the length 2*n (where n is typically 2-3) and the symbol $*$ stands of the convolution operator. Spectral jumps in $\Delta\vec{E}$ result large absolute values in $\vec{S}$. Thereby, the dataset can be divided in several segments between such events. In a following step, the mean and variance are estimated within each segment separately. The variance is then averaged between all segments to obtain a quantitative estimate of spectral fluctuations under a constant external electric field.

Supplementary Fig. S5: Additional observations for the link between SD and QCSE

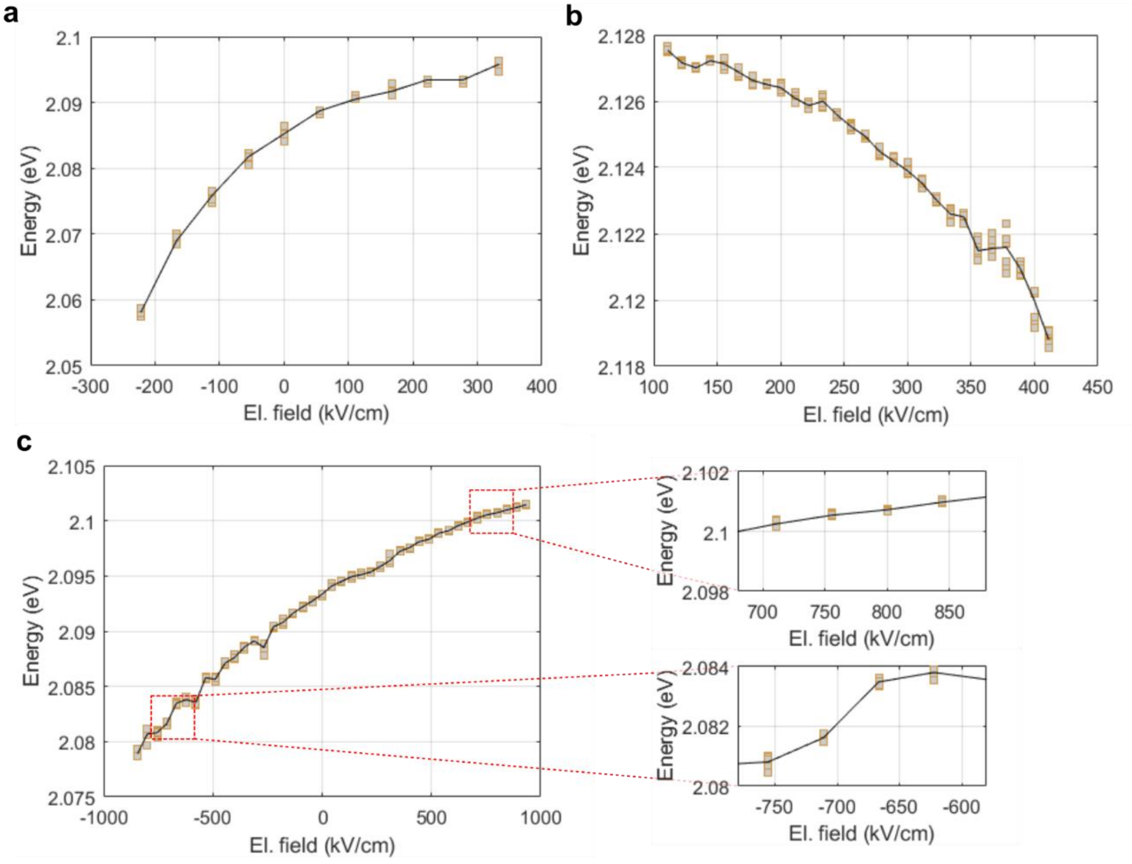

Fig. S5. The extracted energy of the spectral peak (orange squares) analyzed from measurements in which PL spectra of a single QD is measured during a sweep of the electric-field amplitude. A line connecting the mean spectral position for each value of the field is added as a guide to the eye. The extent of spectral fluctuation appears as a dispersion of the orange squares around the black line. In all three cases, an increase in spectral fluctuations occurs as the electric field is driven further away from the QCSE-parabola apex. In the case of panel **c**, we provide two zoom-in insets as it is difficult to visually distinguish spectral fluctuations while presenting the full energy scale.

Supplementary Fig. S6: Analysis of emission linewidth

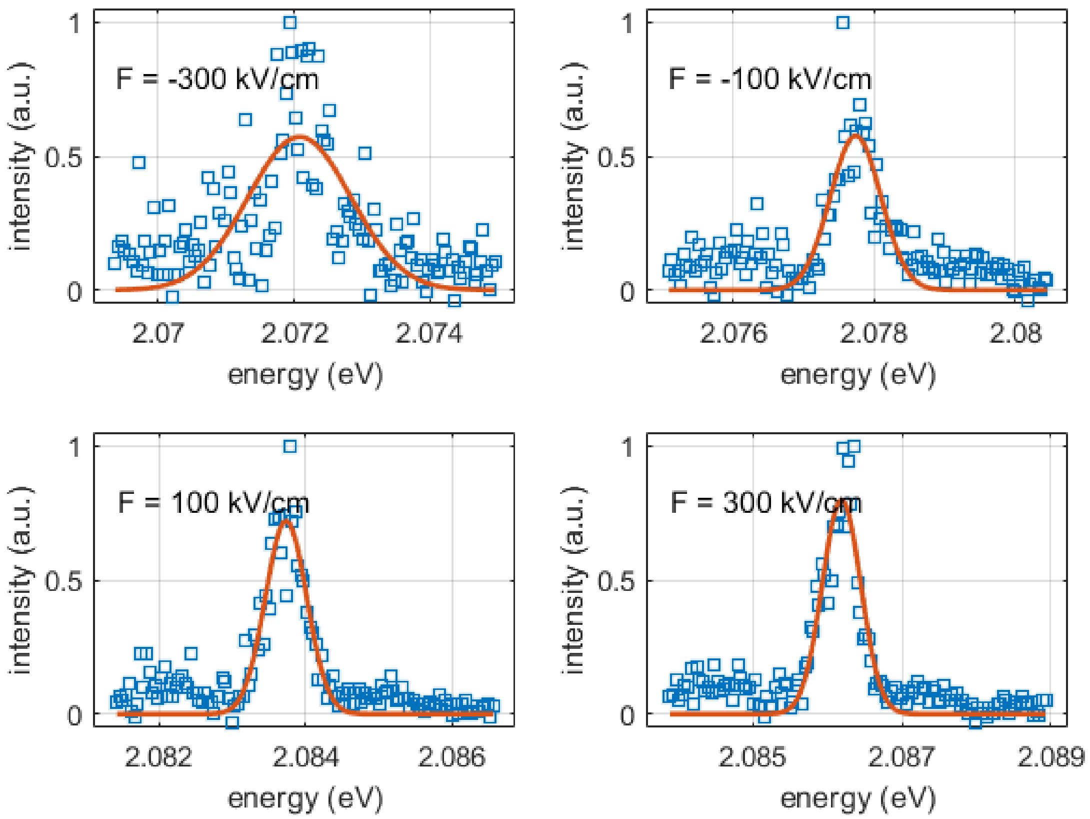

Fig. S6. An example demonstrating the analysis of spectral linewidth for QD1. Panels present the normalized PL spectra for QD1 under four different field-amplitude values (indicated on axes). Each spectrum is fitted with a single Gaussian function (red line). The values for the width parameter Γ, presented in Fig. 4 of the main text, are taken from these fits. We note that outlying high intensities within some spectra are ignored in the fits as they do not belong to the continuous spectral lineshape. While uncertain, we tend to attribute these to systematic noise in the CCD camera rather than to realistic spectral features.

Supplementary note 6: Deriving the dependence of SD on an applied electric field

This section details the numerical model used to fit the dependence of the variance of the PL energy on the externally applied electric field, presented in Fig. 5 of the main text. The relation between the two is formed through the QCSE and the presence of electric-field fluctuation in the micro environment of the QD.

We begin by considering the transition energy from the ground state to the exciton state (E) and its dependence on a general electric field ($\vec{F}$). According to QCSE

$$E = E_0 - \frac{1}{2}\beta\left(\vec{F} - \vec{F}_0\right)^2, \tag{S3}$$

where E_0 is the transition energy in the absence of any electric field and β is the polarizability – a property of the material and the structure of the QD. The model includes a built-in electric-field vector $\vec{F}_0$ that is time independent. The presence of such a field component had been suggested by repeated observation of an offset in the energy maximum in QCSE experiments on multiple type of emitters[7,8]. The environmental electric-field vector ($\vec{F}$) can be further divided into two parts: an externally applied field ($\vec{F}_{ext}$) and a fluctuating component that results from the microscopic environment of the QD ($\delta\vec{F}(t)$). Without loss of generality, we set the external electric field in the $\hat{z}$ direction so that $\vec{F}_{ext} = F_{ext}\hat{z}$, yielding

$$E(t) = E_0 - \frac{1}{2}\beta\left(\delta F_{\parallel} + F_{ext} - F_{0,\parallel}\right)^2 - \frac{1}{2}\beta\left(\delta\vec{F}_{\perp} - \vec{F}_{0,\perp}\right)^2, \tag{S4}$$

where we have separated the fluctuating field into two components - parallel and orthogonal with respect to the external field.

By definition of the statistics for the fluctuating field

$$\langle \delta F_{\parallel} \rangle = 0\,;\ \ \langle \delta\vec{F}_{\perp} \rangle = 0. \tag{S5}$$

Taking the time average of Eq. S*4* under the assumption of ergodicity and plugging in Eqs. S*5*, we obtain

$$\langle E \rangle = E_0 - \frac{\beta}{2}\left[\Delta F_{\parallel}^2 + \langle \delta F_{\parallel}^2 \rangle + \langle \delta\vec{F}_{\perp}^2 \rangle\right]. \tag{S6}$$

Our analysis targets the effect of the fluctuating field on the variance of the transition energy

$$V[E] \equiv \langle (E - \langle E \rangle)^2 \rangle\ = V_0 + \beta^2 \langle \delta F_{\parallel}^2 \rangle \cdot \left(F_{ext} - F_{0,\parallel}\right)^2, \tag{S7}$$

where V_0 contains multiple terms that do not depend on F_{ext} - the parameter varied in our experiments. In the last step, we have assumed that

$$\langle \delta F_{\parallel}^3 \rangle = 0\,, \tag{S8}$$

enforcing reversal symmetry on the fluctuations and

$$\langle \delta F_{\parallel} \delta\vec{F}_{\perp}^2 \rangle = 0\,, \tag{S9}$$

relying on a lack of correlation between the electric-field fluctuations in orthogonal directions. A violation of one of these assumption leads to a term with a linear dependence on $F_{ext} - F_{0,\parallel}$ in Eq. S*7*. Note that while a correlation between orthogonal field components is reasonable on short time scales, it is expected to average out when a charge carrier samples multiple positions and displacement directions in the vicinity of the QD. While the final result of the following analysis, relying on Eq. S9, agrees well with the experimental data, the SNR of the data does not allow us to rule out the presence of an extra linear term in Eq. S7, leading to a more complicated trend in Eq. S11 below.

To simplify the comparison of this model to the experimental results, we note that

$$\frac{\partial\langle E\rangle}{\partial F_{ext}} = \beta\left(F_{ext} - F_{0,\parallel}\right) . \qquad \text{S10}$$

Using the last expression in Eq. S*7*, we achieve the main result of this section,

$$V[E] = V_0 + \left(\frac{\partial\langle E\rangle}{\partial F_{ext}}\right)^2 \cdot \langle\delta F_{\parallel}^2\rangle . \qquad \text{S11}$$

Fig. 5 of the main text and Fig. S7 of the supplementary information present the comparison of the expression in Eq. S*11* to the experimental data of 4 different individual quantum dots. The fit parameters here are V_0 and $\langle\delta F_{\parallel}^2\rangle$. The first term contains multiple contributions due to fluctuations in the field components orthogonal to the external electric field. In addition, noise in the measurement of the PL spectrum also contributes to external-field-independent fluctuations in the PL energy peak, effectively added to V_0.

Supplementary Fig. S7: Numerical modeling of SD for two additional QDs

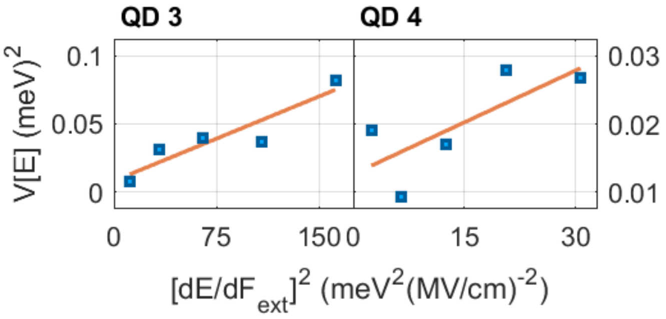

Fig. S7. A quantitative analysis of the relation between SD and QCSE for two additional single-QD measurements. This figure is identical in format to Fig. 5 of the main text. The dependence of slow fluctuations variance (σ_E^2) on the square of the derivative of the energy with respect to the electric field for QD3 (left) and QD4 (right). Linear fits (orange lines) indicate that the simplistic model presented in Eq. (5) of the main text is in a good agreement with our results. $\sqrt{\langle \delta F_z^2 \rangle} = 20\ kV/cm$ (QD3) and $\sqrt{\langle \delta F_z^2 \rangle} = 23\ kV/cm$ (QD4).

Supplementary note 7: The dependence of spectral fluctuations on the power and wavelength of laser excitation

This section investigates the effect of the power and the wavelength of the excitation laser on the observed spectral fluctuations in the emission of individual QDs.

First, a series (30-50) of high-resolution PL spectra was measured for 46 QDs with a temporal resolution of 0.5-2 s under a varying level of laser power (P). Three example datasets are provided in Figs. S8-S10. An autocorrelation-based algorithm is applied to find the spectral shift for each spectrum with respect to the most common peak energy. In order to exclude rare periods in which the intensity 'blinks' off, spectra whose integrated intensity is more than two standard deviations below the mean are discarded. To analyze spectral diffusion, we calculate the energy shifts between consecutive spectra (Figs. S8-S10, bottom left). Since we are interested only in continuous and reversible spectral trends, we discard of outlying spectral jumps in the meV scale (>1.5 standard deviation above or below mean). Finally, the standard deviation of the spectral shifts (σ_E) is calculated for every laser excitation power separately, yielding the graph shown in Figs. S8-S10 (bottom right).

S8

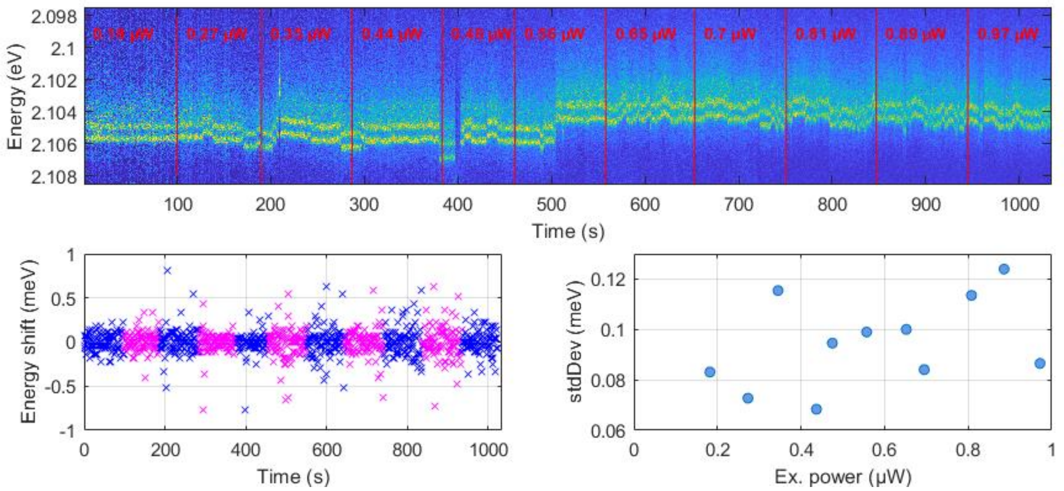

S9

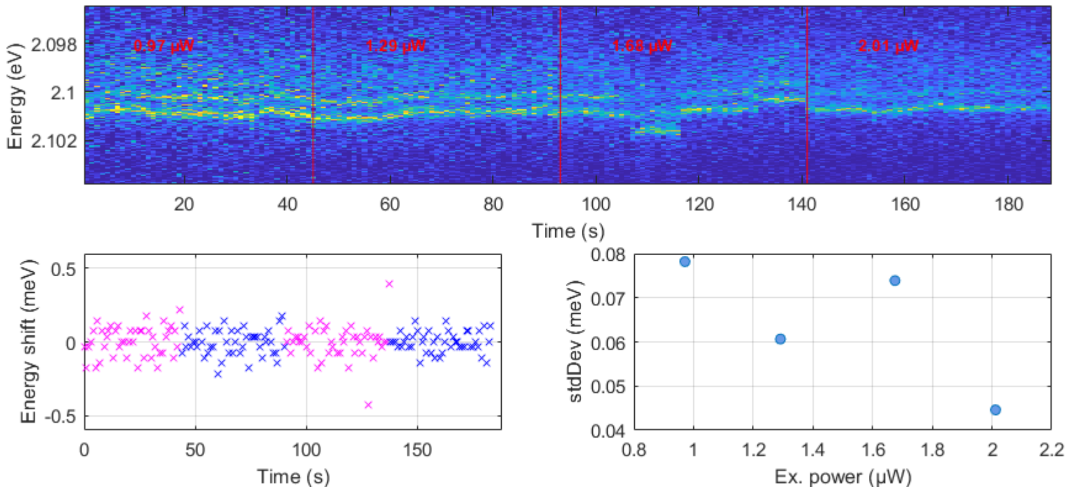

S10

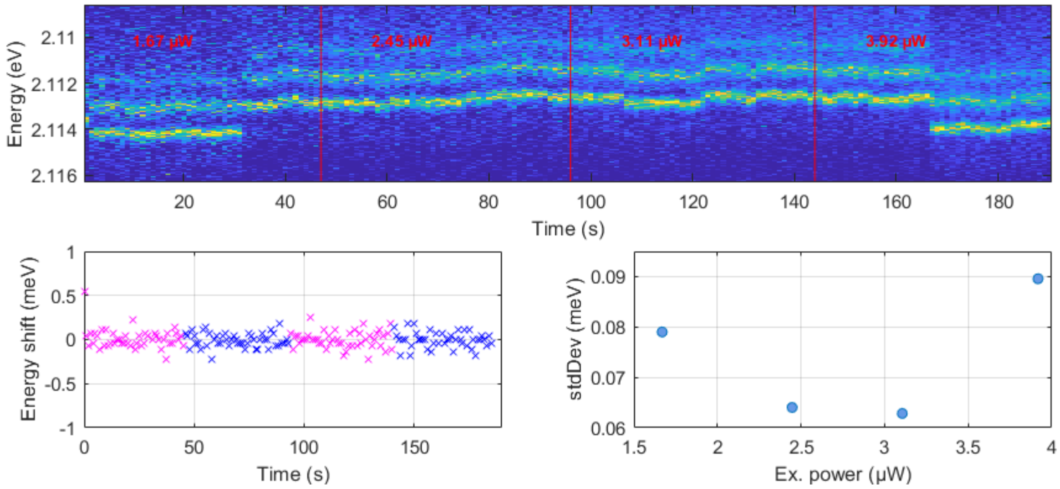

Figs. S8-S10. Top: example dataset 1-3 for a measurement of spectral fluctuations *versus* the power of the excitation laser (indicated in red text), respectively. For a clear comparison between different powers, we normalize the signal in each step according to the excitation power. Bottom: energy shifts between consecutive spectra over the entire measurement time (left). Spectral fluctuations manifest as the spread in the y direction. Interchanging colors serve to highlight the time point in which power is switched. With increasing power, the standard deviation of shifts increases in Fig. S8, decreases in Fig. S9 and shows no systematic trend in Fig. S10.

From these datasets, one can already notice the variation in trend between different QDs. It is therefore important to statistically analyze this data over the ensemble of measured QDs. Fig. S11 presents a histogram for the average change of σ_E with excitation power. Clearly, this parameter distributes rather symmetrically around zero, i.e. for a randomly selected QD, spectral fluctuations are just as likely to increase with P as they are to decrease. To further analyze this data, we manually classify the trends of spectral fluctuation *versus* excitation power (as the ones shown in the bottom right of Figs. S8-S10) for all QDs (colored bars in Fig. S11). Under this classification, most QDs (66%) either do not show a significant change in $\sigma_E(P)$ or do not present a systematic one.

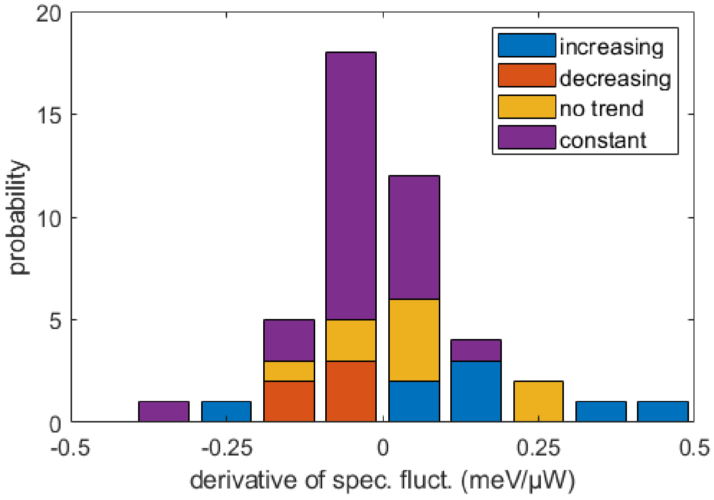

Fig. S11. A histogram of the average derivative in $\sigma_E(P)$ for the 46 QDs measured. Colored bars indicate a manual classification of the graphs into those with increasing (blue), decreasing (red) or constant

(purple) σ_E with increasing excitation laser power. Yellow bars indicate measurements in which no clear and systematic trend was observed.

Finally, we turn our attention to the scale of the trends here. A typical linewidth for the QDs measured in this work is ~ 0.2 meV. The scale of excitation power is 1 µW. Thus, 80% of the QDs display an added spectral noise below one linewidth with a doubling of the excitation power. From these measurements we can confirm that spectral fluctuations are likely not caused by the average power of the laser. This rules out several explanations for the cause of electric-field fluctuations including the detrapping of charge carriers assisted by laser heating of the QD's surrounding.

An equivalent experiment was performed to investigate the effect of the laser excitation wavelength on spectral fluctuations in the PL of single QDs. Here, we measured 10 individual QDs under 2-5 different laser wavelengths (λ_{exc}) in the range of 520-570 nm while the laser power was maintained constant. Two example datasets are presented in Figs. S12 and S13. Using the same analysis procedure as for the laser power-dependent experiments, we evaluate σ_E *versus* λ_{exc} (bottom right). As in the given examples, in all our measurements no significant changes in σ_E with variation of the laser wavelength were detected. While, in the future, this dataset can be expanded for a continuous scan of the excitation wavelength, we believe that the current analysis already strongly indicates that the laser wavelength does not substantially affect spectral fluctuations.

Considering both of the experimental investigations presented in this section together, we conclude that the continuous and reversible trends of spectral fluctuations do not depend on the laser excitation source. As a result, it is more likely that electric field fluctuations are an inherent quantity that is related to the nanocrystals structure (e.g. fluctuation of ligands, charge trapping within the polymer shell) or to their environment (e.g. free or trapped charges in the substrate).

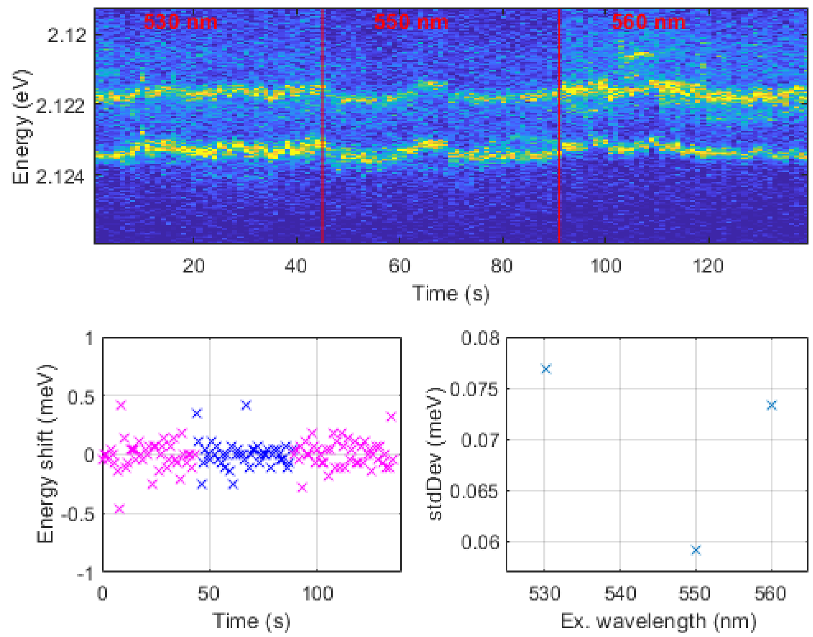

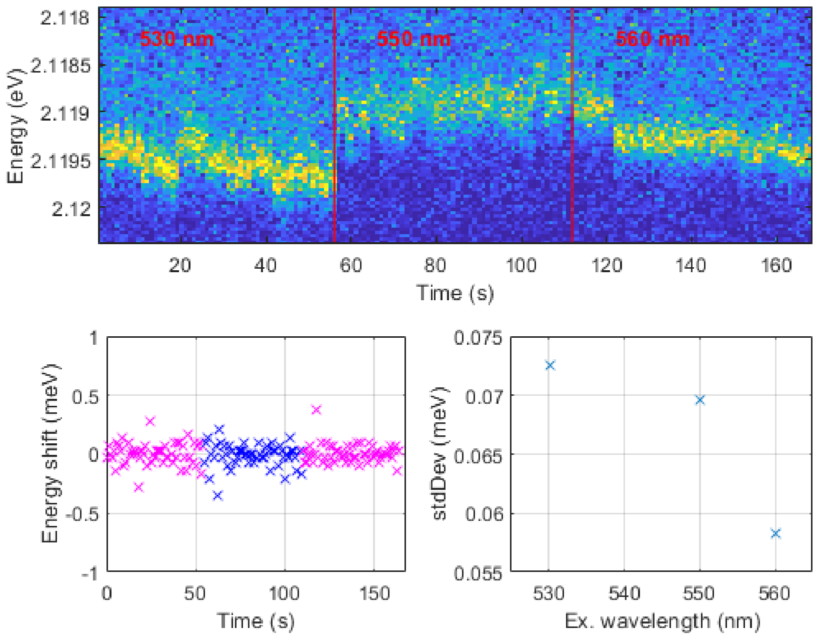

Fig. S12-S13. Top: example datasets 1 and 2 for a measurement of spectral fluctuations versus the wavelength of the excitation laser (indicated in red text), respectively. Bottom: energy shifts between consecutive spectra over the entire measurement time (left). Spectral fluctuations manifest as a spread in the y direction. Interchanging colors serve to highlight the time points in which a switch of wavelength occurs. Changes to σ_E are very small and no clear trend can be established.

References

(1) Huber, S. Multi-Shell Hybrid Nanoparticles From Controlled Polymerizations, Universität Konstanz, 2021.

(2) Carbone, L.; Nobile, C.; De Giorgi, M.; Sala, F. D.; Morello, G.; Pompa, P.; Hytch, M.; Snoeck, E.; Fiore, A.; Franchini, I. R.; Nadasan, M.; Silvestre, A. F.; Chiodo, L.; Kudera, S.; Cingolani, R.; Krahne, R.; Manna, L. Synthesis and Micrometer-Scale Assembly of Colloidal CdSe/CdS Nanorods Prepared by a Seeded Growth Approach. *Nano Lett.* **2007**, *7* (10), 2942–2950.

(3) Coropceanu, I.; Rossinelli, A.; Caram, J. R.; Freyria, F. S.; Bawendi, M. G. Slow-Injection Growth of Seeded CdSe/CdS Nanorods with Unity Fluorescence Quantum Yield and Complete Shell to Core Energy Transfer. *ACS Nano* **2016**, *10* (3), 3295–3301.

(4) De San Luis, A.; Bonnefond, A.; Barrado, M.; Guraya, T.; Iturrondobeitia, M.; Okariz, A.; Paulis, M.; Leiza, J. R. Toward the Minimization of Fluorescence Loss in Hybrid Cross-Linked Core-Shell PS/QD/PMMA Nanoparticles: Effect of the Shell Thickness. *Chemical Engineering Journal* **2017**, *313*, 261–269.

(5) Gumbsheimer, P.; Conradt, F.; Behovits, Y.; Huber, S.; Hinz, C.; Negele, C.; Mecking, S.; Seletskiy, D. V.; Leitenstorfer, A. Enhanced Determination of Emission Fine Structure and Orientation of Individual Quantum Dots Based on Correction Algorithm for Spectral Diffusion. *Journal of Physics D: Applied Physics* **2021**, *54* (15), 155106.

(6) Magid, L. M. Electromagnetic Fields, Energy, and Waves. **1972**.

(6) Empedocles, S. A.; Bawendi, M. G. Quantum-Confined Stark Effect in Single CdSe Nanocrystallite Quantum Dots. *Science* **1997**, *278* (5346), 2114–2117.

(7) Tamarat, Ph.; Gaebel, T.; Rabeau, J. R.; Khan, M.; Greentree, A. D.; Wilson, H.; Hollenberg, L. C. L.; Prawer, S.; Hemmer, P.; Jelezko, F.; Wrachtrup, J. Stark Shift Control of Single Optical Centers in Diamond. *Phys. Rev. Lett.* **2006**, *97* (8), 083002.

(9) Madelung, O. *Semiconductors: Data Handbook*; Springer Berlin Heidelberg: Berlin, Heidelberg, Heidelberg, 2004. https://doi.org/10.1007/978-3-642-18865-7.
